# The extent of involvement of ouabain, hippocampal expression of Na^+^/K^+^-ATPase, and corticosterone/melatonin receptors ratio in modifying stress-induced behavior differs according to the stressor in context

**DOI:** 10.1590/1414-431X2022e11938

**Published:** 2022-07-13

**Authors:** S. Abdelmissih, W.M. Sayed, L.A. Rashed, M.M. Kamel, M.A. Eshra, M.I. Attallah, R.A-R. El-Naggar

**Affiliations:** 1 Department of Medical Pharmacology, Faculty of Medicine, Kasr Al-Ainy, Cairo University, Cairo, Egypt; 2 Department of Anatomy and Embryology, Faculty of Medicine, Kasr Al-Ainy, Cairo University, Cairo, Egypt; 3 Department of Medical Biochemistry and Molecular Biology, Faculty of Medicine, Kasr Al-Ainy, Cairo University, Cairo, Egypt; 4 Department of Basic Medical Science, Faculty of Medicine, King Salman International University, South Sinai, Egypt; 5 Department of Physiology, Faculty of Medicine, Kasr Al-Ainy, Cairo University, Cairo, Egypt; 6 Department of Pharmacology and Toxicology, Faculty of Pharmacy, Misr University for Science and Technology, Giza, Egypt

**Keywords:** Ouabain, Sodium/potassium pump, Corticosterone receptors/melatonin receptors, Hippocampal neurodegeneration, Immobilization, Prolonged darkness

## Abstract

The aim of this study was to assess the effect of two types of stressors, regarding the extent of involvement of ouabain (OUA), hippocampal sodium/potassium ATPase (*NKA*) expression, and the hippocampal corticosterone receptors (*CR*)/melatonin receptors (*MR*) expression ratio, on the behavioral and cardiovascular responses and on the hippocampal cornu ammonis zone 3 (CA3) and dentate gyrus (DG). Thirty adult male Wistar albino rats aged 7-8 months were exposed to either chronic immobilization or a disturbed dark/light cycle and treated with either ouabain or vehicle. In the immobilized group, in the absence of hippocampal corticosterone (CORT) changes, rats were non-responsive to stress, despite experiencing increased pulse rate, downregulated hippocampal sodium/potassium pump, and enhanced hippocampal *CR/MR* expression ratio. Prolonged darkness precipitated a reduced upright attack posture, with elevated CORT against hippocampal *MR* downregulation. Both immobilization and, to a lesser extent, prolonged darkness stress resulted in histopathological and ultrastructural neurodegenerative changes in the hippocampus. OUA administration did not change the behavioral resilience in restrained rats, despite persistence of the underlying biochemical derangements, added to decreased CORT. On the contrary, with exposure to short photoperiods, OUA reverted the behavior towards a combative reduction of inactivity, with unvaried *CR/MR* and CORT, while ameliorating hippocampal neuro-regeneration, with co-existing *NKA* and *MR* repressions. Therefore, the extent of OUA, hippocampal *NKA* expression, and *CR/MR* expression, and subsequent behavioral and cardiac responses and hippocampal histopathology, differ according to the type of stressor, whether immobilization or prolonged darkness.

## Introduction

Stress can evoke a multitude of responses, some of which serve as an adaptation to a perceived threat or ‘stressor', while others might be pathological (1). In other words, stress-provoked behaviors can be either protective or pernicious (2), up to criminal acts. It has been reported that people die from homicide three times more than from war, and homicide is predicted to be among the top 20 causes of death by 2030 (3).

During stress, activation of the hypothalamic-pituitary-adrenocortical axis (HPAA), with release of corticosteroids (CS), assists in increasing energy availability (1). This daytime hormone binds to glucocorticoid receptors (GRs) in the hippocampus, an area that is highly vulnerable to stress (4). Chronic stress exposure induces hippocampal neurodegeneration with consequent disturbance of the neuroendocrine pathway, causing elevated CS release (5). In turn, chronic high levels of CS cause maladaptation to routine challenges by inducing structural neurodegenerative changes in the hippocampus, such as neuronal necrosis (6). Deleterious effects over mood and/or sleep are evident among patients treated with systemic or intra-articular CS (7). Nevertheless, increased CS following chronic stress is not consistent, so that stress can result in either increased or decreased CS levels (8).

Despite extensive research on the involvement of CS as stress hormones, no single neurochemical has been proven specific for stress responses. Hence, another neurohormone, melatonin, evolved as mediator of the stress-triggered behaviors, especially elevated upon stress exposure (9). In a study of hostile human attitudes, a correlation between this night hormone and reduced sleep time was detected (10). Thus, it is not a coincidence that melatonin receptors (MRs) are expressed in adrenal glands where CS are synthesized and released, and that they share common expression sites with GRs in the hippocampus, a brain region that plays a role in sleep regulation and is unique in its neurogenesis, even in adulthood (11).

The understanding of the process became more complicated when the strong association between the energy-dependent sodium/potassium ATPase (Na^+^/K^+^-ATPase “NKA”) and bipolar manic or depressive states, two psychiatric issues triggered by stress (12), was revealed. Recently, chronic stress was implicated in enhancing the protein content of NKA alpha-1 and alpha-2 subunits in cardiac tissue of adult male rats, in the absence of elevated corticosterone (13).

Moreover, digitalis-like compounds (DLCs), negative regulators of NKA, gained attraction in stress research because they share a common site of synthesis and release with stress CS hormones in the adrenal gland and are secreted by the adrenal glands, under the control of the HPAA (14). Among DLCs, ouabain (OUA) was reported to increase along with CORT in brain and adrenals of rats subjected to stress (15). Chronic intraperitoneal administration of a low dose of OUA was able to enhance behavioral performance and promote neuronal proliferation after ischemic brain injuries (16).

To complement previous studies addressing stress-elicited responses, the current study assessed the magnitude of OUA, hippocampal *NKA* expression, and CORT receptors/melatonin receptors (*CR/MR*) expression ratio in modulating stress-associated behavior of adult male rats exposed to two types of stressors (1 h immobilization and disturbed dark/light cycle) and the impact in the underlying histopathological derangement. Secondary goals were to track the involvement of hippocampal *NKA* expression in the dynamics of OUA during stress and investigate the correlation between *NKA* expression and hippocampal receptors' expression ratio of the day and night hormones.

Immobilization stress is an effective model to replicate an array of stress-provoked changes in the rodent model at behavioral, biochemical, and cardiovascular levels, including anxiety and depressive-like behaviors (17), release of corticotropin-releasing hormone (18), and possibly, hypertension and tachycardia (19). Similarly, as normal circadian rhythm has been linked to almost every aspect of life, the behavioral and biochemical aspects of CS and melatonin are especially relevant to our study. By altering day-light phases, we can obtain the variations needed for our investigation. In addition, rodents and humans have a similar pattern of nocturnal peak of melatonin, despite rats being nocturnal animals, unlike humans who are diurnal (20).

## Material and Methods

### Material

**Figure 1:**
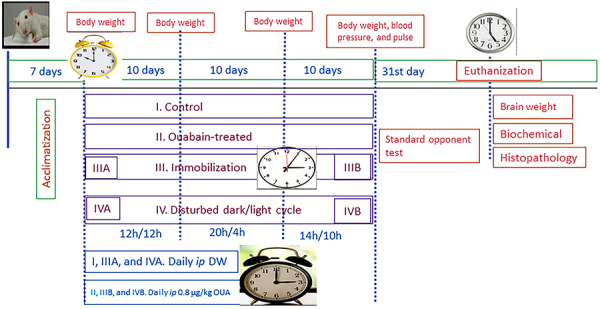
Experimental design. After 7-day acclimatization, adult male Wistar albino rats (7-8 months of age) (N=30) were subdivided into 4 main groups, Control I (n=5), Ouabain-treated II (n=5), Immobilization stress III (n=10), and Disturbed dark/light cycle IV *(*n=10). In Immobilization (III), rats were restrained every other day for 1 h in a restrainer for 30 days. In Disturbed dark/light cycle (IV), rats were subjected to dark/light (12/12 h for the first 10 days), dark/light (20/4 h for the next 10 days), and then dark/light (14/10 h for the last 10 days). Each of the two subgroups III and IV was further subdivided, equally, into either A (untreated, n=5) or B (ouabain-treated, n=5). In groups I, IIIA, and IVA, rats were administered distilled water (DW) *ip* daily at a volume of 0.36 mL/200 g body weight for 30 days; in groups II, IIIB, and IVB, they were given ouabain (OUA) *ip* at a dose of 0.36 μg/200 g body weight daily for 30 days. Animals were weighed initially, and at the 10th, 20th, and 30th days. At day 30, blood pressure and pulse were recorded. At day 31, behavioral assessment was done, followed by euthanization, brains were weighed, and then hippocampi were dissected for biochemical and histopathological assessment.

OUA was purchased as powder (Sigma Chemical Co., USA), freshly dissolved in distilled water, and administered by the intraperitoneal (*ip*) route, at a dosage of 1.8 μg/kg, once daily (12) (Figure 1).

### Animal grouping

Thirty adult male Wistar albino rats (140-290 g) aged 7-8 months, age of social maturation in rats (21), were purchased from the Animal House, Faculty of Medicine, Kasr Al-Ainy, Cairo University (Egypt). Five rats/cage were acclimatized for 7 days before the start of the experiment at the Medical Pharmacology Department, Kasr Al-Ainy, Cairo University. The animals were kept under standard conditions (22±2°C room temperature; 45-50% relative humidity; 12-h light/dark cycle with lights on at 07:30), with free access to food and water, and housed in accordance with the Animal Research Reporting of *In Vivo* Experiments (ARRIVE) 2.0 guidelines. Training sessions, drug administration, and behavioral tests were performed between 10:00 and 17:00. The whole experiment was performed during the summer months (May 15 until June 16) in Cairo, Egypt.

All experimental procedures involving animals were performed at the Faculty of Medicine, Kasr Al-Ainy, Cairo University. All experimental protocols were approved by Cairo University Institutional Animal Care and Use Committee. The guidelines for animal handling and use were in accordance with ARRIVE guidelines (https://arriveguidelines.org).

The rats *(*N=30*)* were allocated to four main groups: Group I, “Control” (n=5), in which rats were administered distilled water *ip* daily at a volume of 0.36 mL/200 g body weight for 30 days; Group II, “OUA-treated group” (n=5) in which rats were administered OUA *ip* at 0.36 μg/200 g body weight (12), daily for 30 days (22); Group III, “Immobilization stress” (n=10), in which rats were restrained every other day for 1 h in a restrainer for 30 days; and Group IV, “Exposed to a disturbed dark/light cycle” (n=10), where rats were subjected to dark/light - 12/12 h for the first 10 days, dark/light - 20/4 h for the next 10 days, and then dark/light cycle of 14/10 h for the last 10 days. The III and IV groups were further subdivided into “Untreated A” and “OUA-treated B” groups. Subgroups IIIA and IVA (5 rats/subgroup) were administered distilled water *ip* daily at 0.36 mL/200 g body weight for 30 days and subgroups IIIB and IVB (5 rats/subgroup) were administered OUA *ip* at 0.36 μg/200 g body weight (12) daily for 30 days. Treatment was given at 15:00, 5 min prior to restraint (23).

### Standard opponent test for behavioral assessment

A standard opponent test was used to assess male aggression in the presence of an unfamiliar male rat put together for a certain time in a new cage. By the end of the study, the rats were kept for 24 h, with no intervention, in the Acclimatization Room in the Medical Pharmacology Department, Kasr Al-Ainy, Cairo University. On the day of the test (day 31), one cage containing a single group (5 rats) was brought into the experimentation room and left for two hours for acclimatization. Then, a male rat was placed alone and unseen by his conspecifics in a novel environment (glass cage with acrylic walls opened at the top, dimensions: 40×40×30 cm). Then, an unfamiliar standard partner of similar weight and age was placed in the glass cage, and both rats were left for one minute prior to video recording, which lasted for 15 min or less if severe attacks or biting was observed in vulnerable regions (belly, neck, and paws) for later scoring. Video recording was made with a FUJIFILM digital camera, Japan (FinePix S5800; 8.0 Megapixel; 10× Optical Zoom). The rat used as a standard opponent during behavioral testing was pre-categorized as dominant by repeated tests with other males and marked with blue streaks on the back. The cages were cleaned between each test to remove any olfactory cues that could affect behavioral reactions. Latency and duration (in seconds) of body sniffing, upright posture, inactivity, and raised head were recorded twice by the same observer and not repeated if variability was ≤2 s. All thirty animals were tested.

### Non-invasive blood pressure and pulse measurements

At day 30, blood pressure and pulse were measured in all thirty rats, using rat-tail cuff sphygmomanometer (PanLab, Spain).

### Percent changes in body weight and brain-to-body weight ratio

Before starting the experiment (initial) and at 10-day intervals (10, 20, and 30 days), the rats were weighed; body weights are reported in grams, and the percent body weight changes were calculated by the end of 10, 20, and 30 days, as follows: percent body weight change = [(body weight (g) at days 10, 20, or 30 minus initial body weight (g)), divided by initial body weight (g)] × 100.

At the end of the study (on day 31), after performing the behavioral analysis, rats were sacrificed by decapitation at 17:00, the brains were removed and weighed, and the percent brain-to-body weight ratio was calculated by dividing the brain weight (g) by the body weight on day 30 (g).

### Enzyme-linked immuno-absorbent assay of hippocampal corticosterone

The hippocampus from all 30 animals, randomly chosen for right or left hemispheres, was homogenized and used for biochemical assessments. CORT level was assessed in brain homogenate. After two freeze-thaw cycles, the homogenate was centrifuged for 5 min at 5000 *g* and 2-8°C. The supernatant was removed and assayed using an ELISA kit (Cusabio, CSB-E07014r, USA).

### Quantitative real-time polymerase chain reaction (qRT-PCR)

Quantitative RT-PCR was utilized for quantitative investigation of gene expression of melatonin receptors, CORT receptors. and *Na* ^+^/K^+^-ATPase in all 30 animals. The entire RNA was isolated from hippocampal tissue using SV Total RNA Isolation System, following the manufacturer's guidelines (Promega, USA). The levels and purity of RNA were assessed with an ultraviolet spectrophotometer and complementary DNA (cDNA) was composed utilizing SuperScript ΙΙΙ First-Standard Synthesis System (#K1621, Fermentas, USA). Then, the reverse transcriptase master mix consisting of 2 μL of 10X RT buffer, 4 μL of 25 mM MgCl_2_, 2 μL of 0.1 M DTT and 1 μL of SuperScript^®^ ΙΙΙ RT (200 U/μL) was added to the mix and incubated at 25°C for 10 min, followed by 50 min at 50°C. This was followed by amplification of RT-PCR *(*Applied Biosystems, software version 3.0, StepOne^TM^, USA). The reaction contained SYBR Green Master Mix (Applied Biosystems). Gene-specific primer pairs (Table 1) were calculated with Gene Runner Software (Hasting Software, Inc., USA) from RNA sequences of GenBank. Quantitative RT-PCR was performed in a 25-μL reaction volume composed of 2X SYBR Green PCR Master Mix (Applied Biosystems), 900 nM of each primer, and 2 μL of cDNA. Data from real-time assays were calculated using v1.7 sequence detection software (PE Biosystems, USA). The relative expression of the investigated gene mRNA was measured using the comparative Ct method. All measurements were normalized to beta-actin (control housekeeping gene) and reported as fold-change over contextual concentrations detected in the groups.

**Table 1:** Gene-specific primer pairs for quantitative real-time PCR of hippocampal Na^+^/K^+^-ATPase, corticosterone receptors, and melatonin receptors.

Gene of Interest	Forward primer sequence	Reverse primer sequence
Beta-actin	5′-GGTCGGTGTGAACGGATTTGG-3′	5′-ATGTAGGCCATGAGGTCCACC-3′
Corticosterone receptors	5′-TTCGAAGGAAAAACTGCCCAG-3′	5′-CGAGCTTCAAGGTTCATTCCA-3′
Melatonin receptors	5′-CGTGGTGGACATCCTGGG-3′	5′-CGAGGTCTGCCACAGCTAAACT-3′
Na^+^/K^+^-ATPase	5′-CAGTTCACCAACCTCACCTTG-3′	5′-CACTGTACCCAATGTTCTCACCA-3′

Beta-actin was used as the control housekeeping gene.

### Histopathology of rat hippocampus

Dissected right and left hippocampi were fixed in 10% formaldehyde for 24 h. Tissue preparation for H&E staining was performed in three stages: deparaffinization, hydration, and staining. Fixed tissues were dehydrated through ascending grades of ethanol to absolute ethanol. They were cleared in xylene, infiltrated, and embedded in paraffin wax. The sections were dewaxed in xylene and rehydrated through descending grades of ethanol to water. Sections were cut at 4-5 µm thickness. The slides were stained with filtered Harris's hematoxylin for 1 min, rinsed with tap water, immersed in 10% eosin stain for 1-2 min, and rinsed with tap water again. Then, slices were subjected to dehydration in ascending concentrations of alcohol solutions (50, 70, 80, 95% ×2, 100% ×2). The resulting slides were then viewed under a light microscope.

### Ultrastructural preparation of rat hippocampus

Some hippocampal specimens were put immediately in 3% glutaraldehyde for 24 h, washed in phosphate-buffered saline (PBS), and post-fixed in 1% osmium tetroxide. After fixation, specimens were dehydrated and embedded in epoxy resins. Semi-thin sections (1 μm) were stained with toluidine blue. Ultrathin sections (50-60 nm) were stained with uranyl acetate and lead citrate and then examined and photographed using a JEOL transmission electron microscope (JEM-1400 Flash TEM, JEOL USA) at a suitable electron magnification. Images were captured by a CCD optronic camera model AMT, 1632×1632-pixel format as the side mount configuration.

### Histomorphometry measurements

The thickness (mm) of the pyramidal layer of CA3 and the granular layer of the DG of the hippocampus were measured in hematoxylin and eosin-stained sections using a Leica Qwin 500 LTD image analyzer computer system (Software Qwin 500, UK).

### Statistical analysis

Data were coded and entered using the statistical package SPSS (IBM, USA) version 25. Data are reported as means±SD for quantitative variables. Comparisons were carried out using one-way analysis of variance (ANOVA) with a *post hoc* multiple comparisons test for normally distributed quantitative variables, while the non-parametric Kruskal-Wallis test was used for comparison of non-normally distributed quantitative variables with Bonferroni correction. Correlations between quantitative variables were performed using the Spearman correlation coefficient. Differences were considered statistically significant when the P value was <0.05. A sample size of 5 rats in each group was calculated to achieve a *post hoc* power of study of 100%, using the online calculator Clincalc (https://clincalc.com/stats/Power.aspx).

## Results

### Behavioral assessment of the standard opponent test

**Figure 2:**
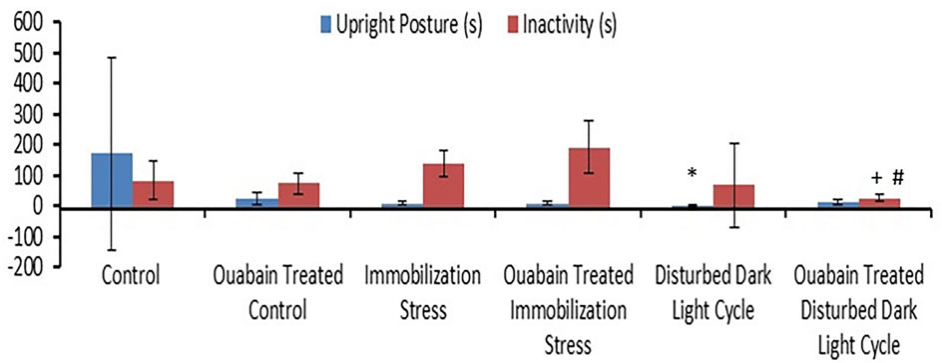
Total duration of upright posture (s) and inactivity (s) in the different study groups. Data are reported as means±SD. *P<0.05 compared to the control group; ^+^P<0.05 compared to the untreated immobilization group; ^#^P<0.05 compared to untreated rats exposed to disturbed dark/light cycle (Kruskal-Wallis test).

Rats submitted to one-hour immobilization (IIIA) did not present behavioral changes, as demonstrated by non-significant changes in total duration for upright posture and inactivity compared to the control rats (I). After 30 days of daily OUA injections to restrained rats (IIIB), the lack of behavioral expression persisted relative to the control (I) and the ouabain-treated (II) groups (Figure 2).

Interestingly, exposure to short photoperiods (IVA) caused a significant decrease in the total duration of upright posture compared to control animals (I) (3±2.45 *vs* 170.2±314.61 s, P=0.03), while total duration of inactivity remained unchanged (Figure 2).

Moreover, OUA treatment of rats in the prolonged darkness condition (IVB) caused a significant decrease in the total inactivity duration (25.2±12.7 s) compared to the untreated rodents, whether they were in the immobilization (IIIA) (139.4±42.72 s; P=0.004) or disturbed dark/light cycles status (IVA) (192.4±138.34 s, P=0.003). However, there was no significant behavioral difference when the OUA-treated rats (II) were compared to the control ones (I), regarding total duration of upright posture and inactivity (Figure 2).

Other tracked behavioral aspects, including latencies (s) of body sniffing, upright posture, inactivity, and raised head, as well as duration (s) of body sniffing and raised head, did not show significant changes among all study groups. Data not shown.

### Blood pressure and pulse

At day 30, blood pressure (mmHg) was not significantly different among study groups. Pulse (bpm) was significantly faster only in immobilized rats (IIIA) compared to the control (I) as well as the OUA-treated rodents (II) (340.4±7.3 *vs* 328.2±2.86 and 328.4±3.44 bpm, respectively, P<0.002) (Table 2).

**Table 2:** Blood pressure (BP) (mmHg) and pulse (bpm) of adult male Wistar albino rats (7-8 months of age).

	Control	OUA-treated	Immobilization stress	OUA-treated immobilization	Disturbed dark/light cycle	OUA-treated disturbed dark/light cycle
Systolic BP	91.8±5.5	88.6±5.73	90.4±7.83	91.2±4.15	86.2±5.85	86.8±4.44
Diastolic BP	68.8±5.36	68.4±5.18	68.6±4.62	68.2±3.42	68.6±3.85	68.6±3.21
Pulse	328.2±2.86	328.4±3.44	340.4±7.3*^+^	332.4±3.65	330.4±3.29	331.6±3.36

Data are reported as means±SD for n=30 rats. *P<0.002 compared to the control group; ^+^P<0.002 compared to ouabain (OUA)-treated group (ANOVA).

### Relative gene expression of *NKA*, *CR*, *MR*, and *CR/MR* ratio

Differential biochemical effects on relative hippocampal sodium/potassium pump expression were found depending on the type of stressor. One-hour alternate-day restraint for 30 days (IIIA) resulted in substantial biochemical changes, culminating in increased HR, despite behavioral non-responsiveness, manifested as downregulation of hippocampal *NKA* expression compared to both the control (I) and the OUA-treated groups (II) (0.27±0.15 *vs* 1.01±0.01 and 1.02±0.03, respectively; P<0.001) against an increased hippocampal *CR/MR* expression ratio (23.38±11.39 *vs* 1.00±0.04 and 1.02±0.04, respectively; P<0.001), most likely owing to higher *CR* expression (6.94±1.50 *vs* 1.01±0.01 and 1.03±0.04, respectively; P<0.001) *vs* lower *MR* expression (0.33±0.10 *vs* 1.01±0.03 and 1.01±0.02, respectively; P<0.001).

Surprisingly, despite showing a submissive posture, rats submitted to 30 days of altered dark/light cycles, at 10-day intervals (IVA), displayed only a significant reduction in hippocampal *MR* expression compared to both the control (I) and OUA-treated animals (II) (0.73±0.11 *vs* 1.01±0.03 and 1.01±0.02, respectively, P<0.001), with unchanged *NKA*, *CR*, and *MR* expression, as well as *CR/MR* ratio. This prolonged darkness-related *MR* downregulation was still significantly bypassed by that detected in the untreated immobilization model (IIIA) (P<0.001).

OUA treatment resulted in discrete behavioral alterations, depending on the type of stressor, with a variable degree of biochemical derangements. In treated-immobilization stress (IIIB), maintaining behavioral resilience, OUA did not change the former biochemical changes, relative to both the controls (I) and OUA-treated group (II), in terms of significantly downregulated *NKA* expression (0.18±0.06; P<0.001) against significantly increased *CR/MR* expression ratio (31.57±8.94; P<0.001), owing to upregulated *CR* (8.18±1.35; P<0.001) *vs* downregulated *MR* (0.27±0.05; P<0.001). Despite an apparent biochemical exacerbation in OUA-treated immobilized rats (IIIB), the variations did not attain significant levels, compared to the untreated immobilized co-peers (IIIA).

Furthermore, chronic OUA administration to rats in short photoperiods (IVB) aggravated the biochemical reaction, more than with the sole exposure to disturbed dark/light cycle model (IVA), as evidenced by the emergence of a significant downregulation of *NKA* expression, relative to the controls (I) and OUA-treated group (II) (0.66±0.13; P<0.001) against a significant increase in *CR* expression (2.97±0.79; P=0.015 and P=0.017, respectively). This was, invariably, associated with significant *MR* downregulation (0.70±0.10; P<0.001), but lacked a significant impact on the *CR/MR* ratio. Such outstanding modifications were much lower than those attained by restrained rats, whether treated (IIIB) or not (IIIA) (P<0.001).

**Figure 3:**
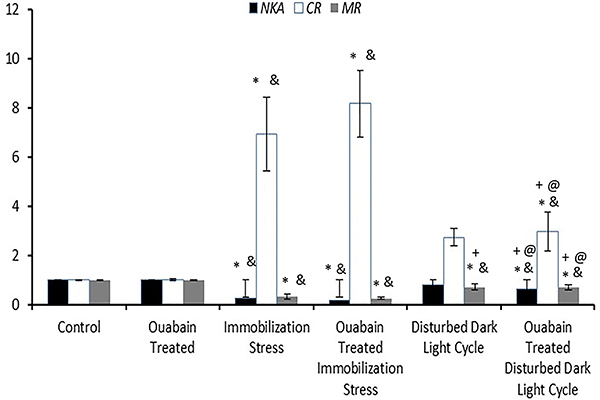
Relative gene expression of hippocampal Na+/K+-ATPase (*NKA*), corticosteroid receptors (*CR*), and melatonin receptors (*MR*) to the housekeeping gene beta-actin of the study groups. Data are reported as means±SD. *P<0.05 compared to the control group; ^&^P<0.05 compared to ouabain-treated group; ^+^P<0.05 compared to untreated immobilization group; ^@^P<0.05 compared to ouabain-treated immobilization group (ANOVA).

**Figure 4:**
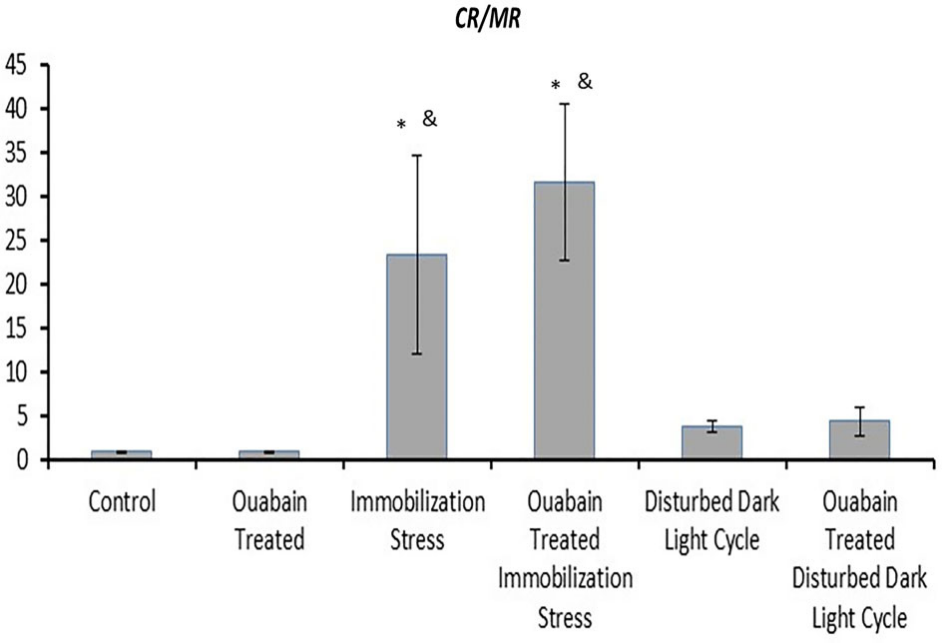
The ratio between relative expressions of hippocampal corticosterone receptors to melatonin receptors (*CR/MR*) to the housekeeping gene beta-actin of the study groups. Data are reported as means±SD. *P<0.05 compared to the control group; ^&^P<0.05 compared to ouabain-treated group (ANOVA).

One month of daily OUA treatment to rats (II) hardly modified biochemical outcomes for the studied hippocampal *NKA*, *CR, and MR* expressions or *CR/MR* expression ratio, relative to control rats (I), consistent to its negligible effect on rats' behavior and cardiovascular indices (Figures 3 and 4).

### Correlation between behavioral aspects and biochemical parameters in hippocampus

**Figure 5:**
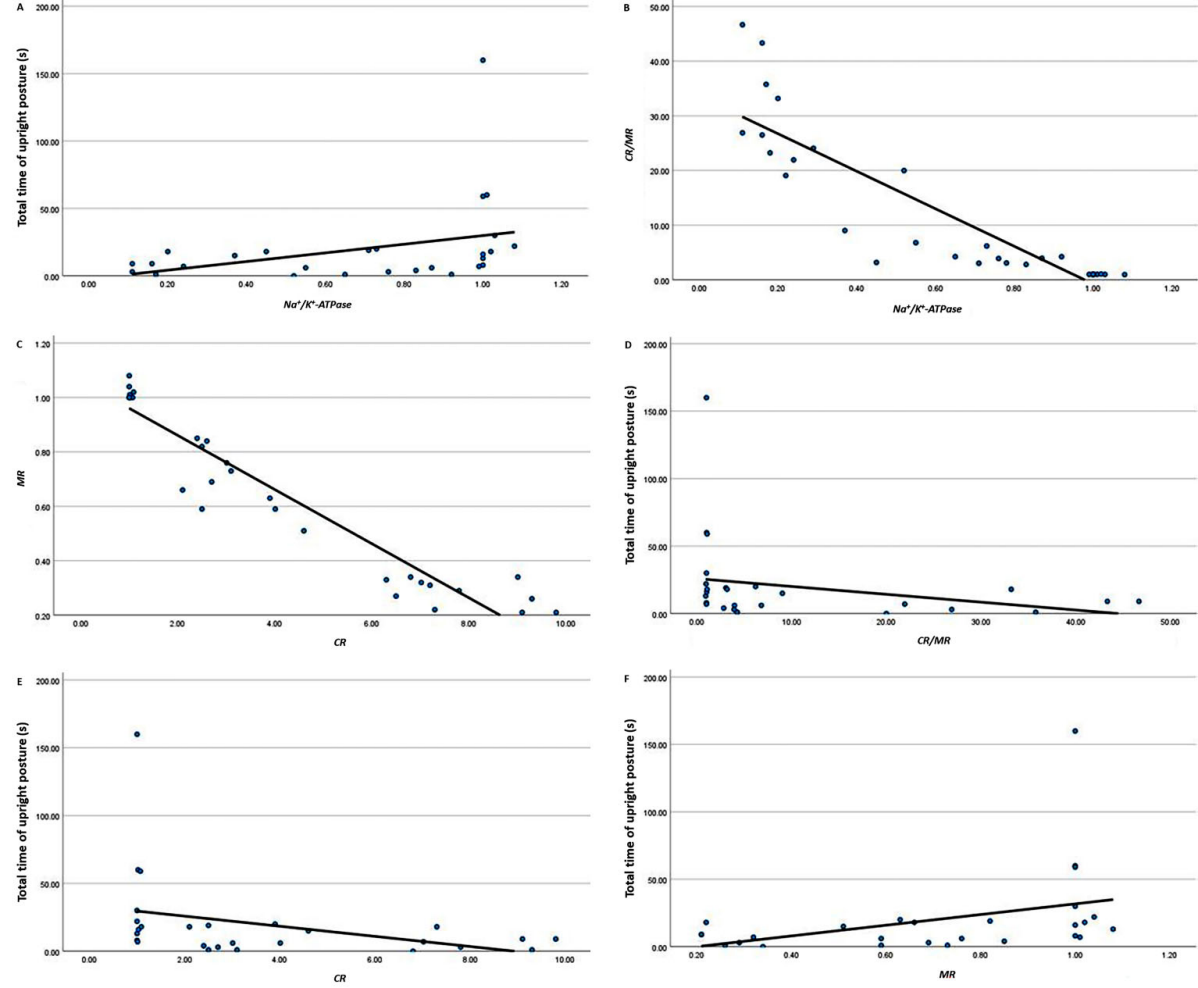
Correlations between **A**) sodium/potassium pump relative gene expression and duration of upright posture (r=0.487, P*=*0.010); **B**) Relative gene expression of Na^+^/K^+^-ATPase and of hippocampal corticosterone receptors to melatonin receptors (*CR/MR*) relative expression (r=-0.931, P=0.001); **C**) Relative *CR* expression and relative *MR* expression (r=-0.938, P=0.001); **D**) duration of upright posture and hippocampal *CR/MR* relative expression ratio (r=-0.477, P=0.012); **E**) duration of upright posture and hippocampal *CR* relative expression (r=-0.508 and P=0.007); **F**) duration of upright posture and relative expression of *MR* (r=0.451, P=0.018) (n=30). Spearman correlation.

Gene expression of the sodium/potassium pump was fairly and positively correlated with the duration of the upright attack posture (s) (r=0.487, P=0.010) (Figure 5A), while the former was negatively correlated with the hippocampal *CR/MR* expression ratio (Figure 5B) (r=-0.931, P=0.001), in addition to the negative correlation between *CR* and *MR* gene expression (r=-0.938, P=0.001) (Figure 5C). This was confirmed by negative correlations found between the duration of upright posture and the hippocampal *CR/MR* expression ratio as well as *CR* expression; the correlation was stronger for the latter (r=-0.477, P=0.012; r=-0.508, P=0.007, respectively) (Figure 5D and E, respectively) *vs* the slight, positive correlation between the upright posture duration and *MR* expression (r=0.451, P=0.018) (Figure 5F).

No correlation was found between the other recorded behavioral features (latencies and durations of body sniffing, inactivity, and head up, as well as latency of upright posture) and the expressions of hippocampal *NKA*, *CR*, and *MR*, and hippocampal *CR/MR* expression ratio. Data are not shown.

### CORT level in hippocampal homogenate

Hippocampal content of CORT (mcg/mL) was not uniform among rats subjected to the two different stressors. In restrained rats (IIIA), despite showing a significant hippocampal *CR* upregulation, hippocampal CORT did not show such a significant path, in line with the indifferent behavior of rats in the standard opponent test. In contrast, exposure to prolonged darkness (IVA) significantly increased hippocampal CORT, relative to both the control (I) and OUA-treated rodents (II) (51.4±2.3 *vs* 42.8±2.28 and 42.8±2.77, respectively, P<0.001), with unchanged *CR*, accompanying the submissive phenotype of rodents.

OUA treatment resulted in differential effects on hippocampal CORT, according to the stressor in context, ranging from significant inhibition in immobilized rats (IIIB), compared to the controls (I) and OUA-treated groups (II) (37.6±2.61, P=0.023), while showing no evident behavioral responsiveness, to no change in rats under short photoperiods (IVB), despite a seemingly hostile attitude.

**Figure 6:**
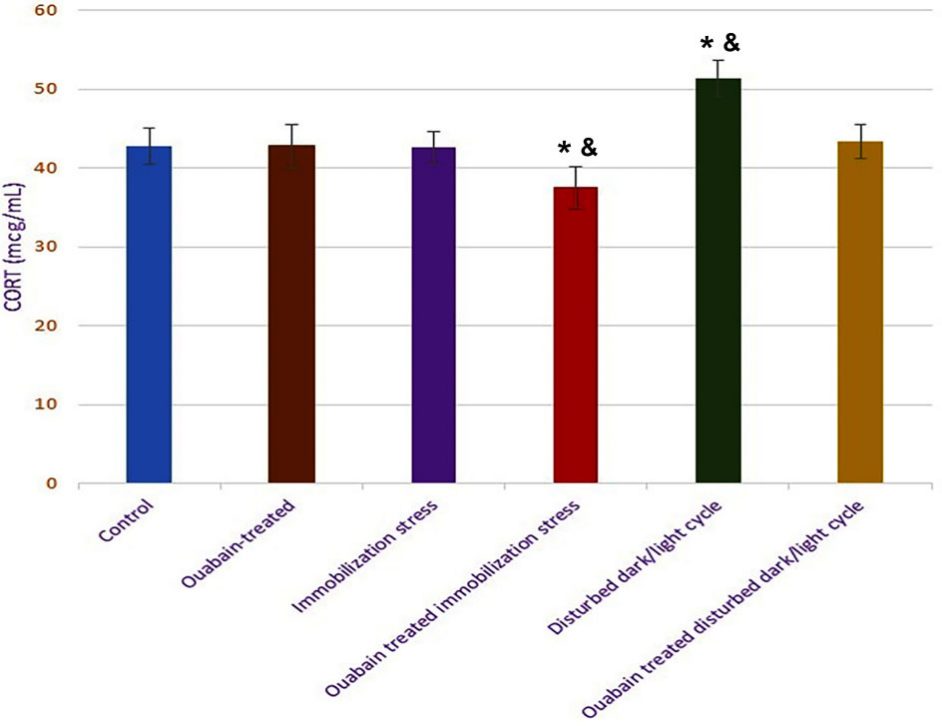
Hippocampal corticosterone (CORT) level (mcg/mL) of study groups. Data are reported as means±SD. *P<0.05 compared to the control group; ^&^P<0.05 compared to ouabain-treated group (ANOVA).

In the OUA-treated group (II), hippocampal CORT was not different from control rats (I), matching their unaffected behavior, cardiovascular, and biochemical parameters (Figure 6).

### Histopathological results

**Figure 7:**
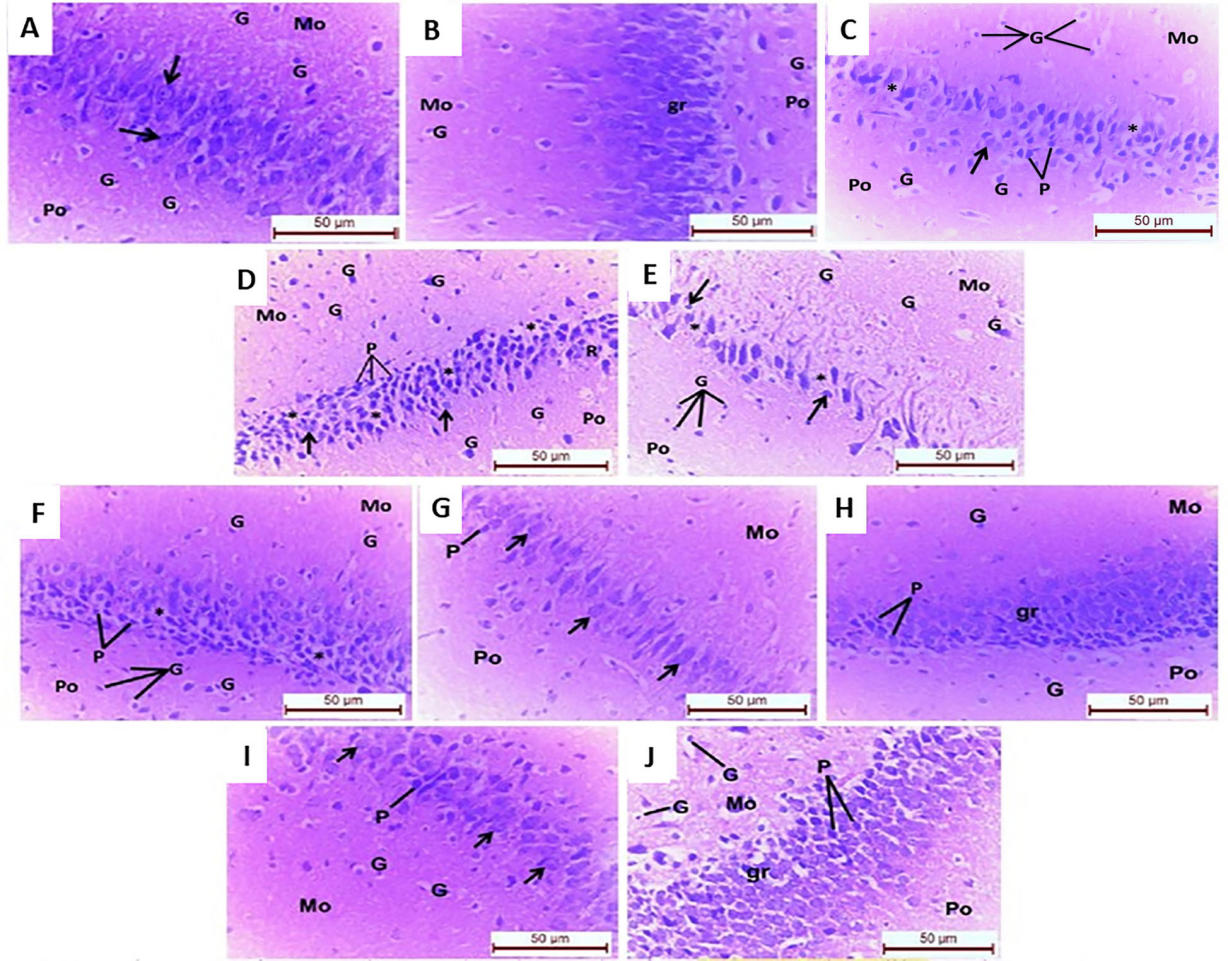
Photomicrographs of hematoxylin and eosin-stained sections from rat hippocampus (7-8 months age) of the study groups. Control and ouabain-treated groups revealing (**A**) normal CA3 zone showing layers of large pyramidal cells (arrows) and glial cells (G) in the molecular (Mo) and polymorphic (Po) layers, (**B**) normal granular cells (gr) in the dentate gyrus with scattered glial cells (G) appear in the molecular (Mo) and polymorphic (Po) layers. Immobilization-stress displaying (**C**) degenerated large pyramidal cells in CA3 zone; cytoplasmic vacuolation (*), pyknotic (P), or karyolitic (arrows) nuclei. Numerous glial cells (G) are also seen within the molecular (Mo) and polymorphic (Po) layers, (**D**) dentate gyrus exhibiting pyknotic (P) and karyolitic (arrows) nuclear alterations and vacuolated (*) or rarefied (R) cytoplasm of granular cells. Several glial cells (G) are also noted in the molecular (Mo) and polymorphic (Po) layers of the dentate gyrus. Disturbed dark/light cycle displaying (**E**) a milder degeneration of pyramidal cells in CA3 zone; pyknotic nuclei (arrows) and vacuolated cytoplasm (*). Multiple glial cells (G) are also seen within molecular (Mo) and polymorphic layers (Po), (**F**) the dentate gyrus exhibiting areas of cytoplasmic vacuolation (*) and hyperchromatic small nuclei (P) of granular cells. The glial cells (G) appear numerous in molecular (Mo) and polymorphic (Po) layers of the dentate gyrus. Immobilization-ouabain revealing (**G**) pyramidal cells with apparently normal architecture; vesicular nuclei (arrows) except for scattered pyknotic nucleus (P). Note the molecular (Mo) and polymorphic (Po) layers with CA3 zone, (**H**) mostly normal granular cells (gr) apart from few pyknotic nuclei (P). The molecular layer (Mo) and polymorphic layer (Po) of dentate gyrus demonstrating scattered glial cells (G). Prolonged darkness-ouabain showing (**I**) apparently normal pyramidal cells with vesicular nuclei (arrows) except for scattered darkly stained nuclei (P), (**J**) preserved granular cells (gr), molecular layer (Mo) and polymorphic layer (Po) of dentate gyrus, except for few hyperchromatic nuclei (P) (×400, scale bar: 50 μm; n=30).

Light microscope examination of hematoxylin/eosin-stained hippocampal sections from control rats (I) demonstrated a normal structure of the cornu ammonis zone 3 (CA3) of the hippocampus proper and the dentate gyrus (DG). The pyramidal layer, one of the three layers of CA3, revealed loosely crowded layers of large cells, each with nearly triangular vesicular nuclei with flimsy cytoplasm. The molecular and polymorphic layers of the CA3 zone were composed of scattered glial cells (Figure 7A). The DG was formed by three layers that were arranged into polymorphic, granular, and molecular layers from the outside to the inside. The granular cell layer displayed a crowded appearance with nearly rounded prominent nuclei and little cytoplasm. The polymorphic and molecular layers showed few glial cells (Figure 7B).

Sections from the hippocampus of OUA-treated rats (II) were not different from the control ones (I) and displayed the same light microscopic features.

Sections from the restrained group (IIIA) demonstrated marked degeneration of the large pyramidal cells of the CA3 zone in the form of vacuolated cytoplasm and nuclear degeneration (pyknosis and karyolysis). Multiple glial cells were also observed within both the molecular and polymorphic layers of the CA3 zone (Figure 7C). The granular cells of the dentate gyrus exhibited degenerative changes in the form of either shrunken (pyknotic) or karyolitic nuclei as well as vacuolated or rarefied cytoplasm. Multiple glial cells appeared either in the molecular layer or the polymorphic layers of the DG (Figure 7D). Similar changes were detected in the group submitted to disturbed dark/light cycle (IVA), although to a lesser extent than the chronic restraint stress (IIIA) (Figures 7E and F).

OUA treatment of the immobilization stress group (IIIB) resulted in moderate improvement of the degenerated CA3 zones and DG. The pyramidal cells displayed nearly normal architecture with vesicular nuclei apart from a few darkly stained nuclei, in addition to apparently normal molecular and polymorphic layers of the CA3 zone (Figure 7G). There was also moderate improvement of the histological structure of the DG, in which the granular neurons revealed seemingly normal architecture apart from a few shrunken nuclei, and the molecular and polymorphic layers were mostly normal and showed glial cells (Figure 7H).

OUA treatment of rats exposed to modified dark/light periods (IVB) resulted in a greater improvement in the form of preservation of most of the pyramidal cells with apparently normal molecular and granular layers, except for scattered darkly stained nuclei (Figure 7I). Additionally, the granular neurons in the DG exhibited nearly normal architecture as well as molecular and polymorphic structures except for few hyperchromatic nuclei (Figure 7J).

### Ultrastructural results

**Figure 8:**
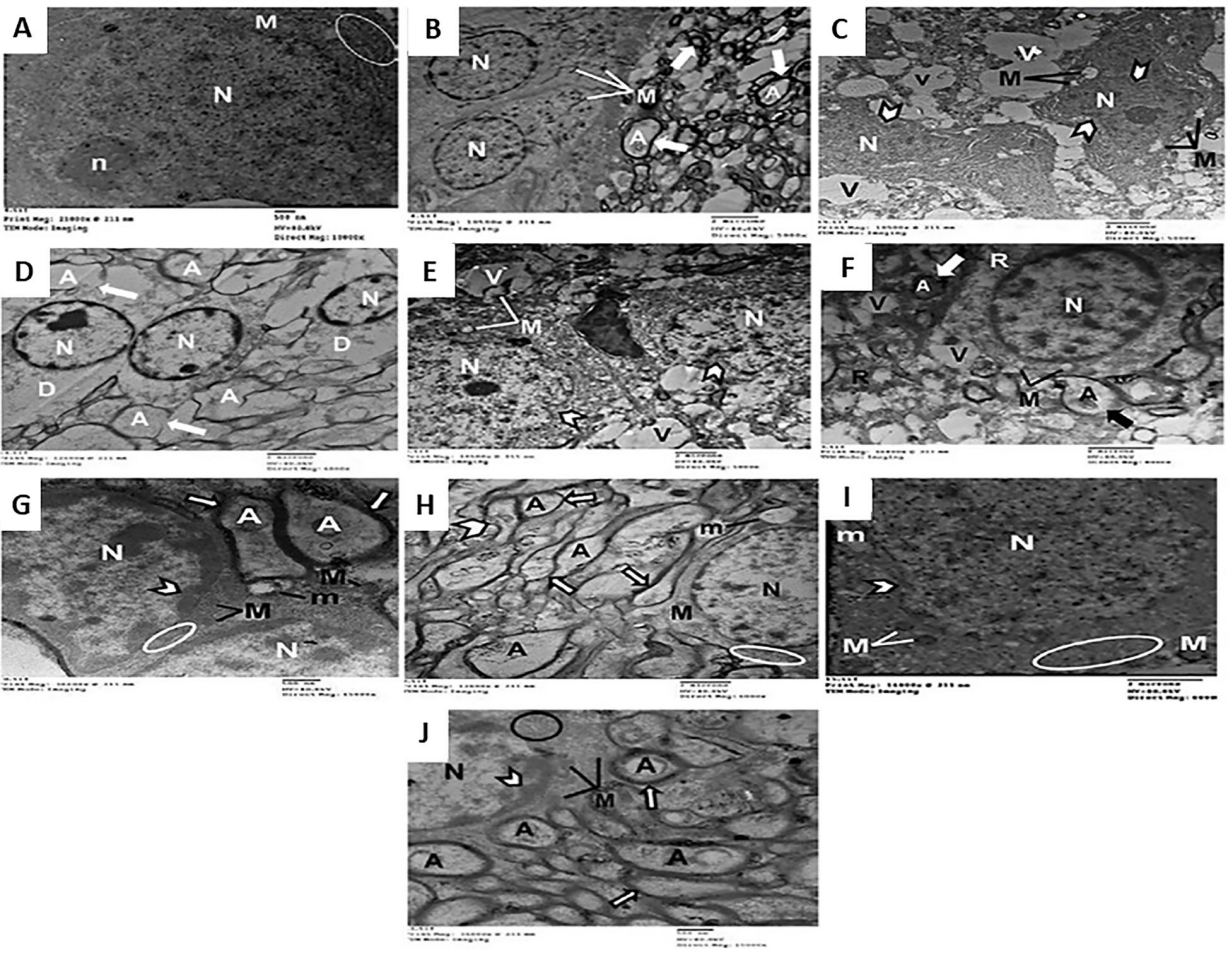
Electron micrographs of ultrathin sections of rat hippocampus (7-8 months age). Control and ouabain-treated groups revealing (**A**) pyramidal cell from CA3 zone revealing normal structure, intact organelles within scanty cytoplasm, mitochondria (M), and rough endoplasmic reticulum (circle). Large triangular nucleus (N) with dispersed distribution of chromatin and prominent nucleolus (n) are also seen (×10,000; scale bar: 500 nm), (**B**) granular cells displaying well circumscribed mitochondria (M) and prominent nuclei (N) with well-defined nuclear envelope. The surrounding neuropil showing myelinated axons (A) with regular compact myelin sheath (arrows) (×5000; scale bar: 2 µm). Immobilization-stress group (**C**) with pyramidal cells demonstrating wide areas of cytoplasmic vacuolation (V) and swollen mitochondria (M) with disturbed cristae. The nucleus (N) exhibited highly condensed chromatin as well as indentation of nuclear envelope (arrowheads) (×5000; scale bar: 2 µm), (**D**) granular cells revealing large areas of degenerated cytoplasm (D), margination of the nuclear chromatin (N), as well as destruction (arrows) of myelin sheath of the axons (A) (×6000; scale bar: 2 µm). Disturbed dark/light cycle group (**E**) vacuolated cytoplasm (V), mitochondria with disturbed cristae (M), and indented nuclear envelop (arrows) of nucleus (N) displaying scattered areas of condensed chromatin (×5000; scale bar: 2 µm), (**F**) granular cell revealing either rarefaction (R) or vacuolation (V) of the cytoplasm as well as disturbed cristae within the mitochondria (M). Margination of chromatin within the nucleus (N) and destruction (arrows) of myelin sheath of the axons (A) are also noted (×8000; scale bar: 2 µm). Immobilization-ouabain group (**G**), pyramidal cells revealing regular nucleus with minimal condensed chromatin (arrowhead), apparently normal mitochondria (M) except for few swollen (m) and intact rough endoplasmic reticulum (circle). The surrounding neuropil showing regular compact myelin sheath (arrows) of the axons (A) (×15,000; scale bar: 500 nm), (**H**) apparently normal mitochondria (M), and rough endoplasmic reticulum (circle) within the granular cell cytoplasm apart from one dilated mitochondrion with ruptured cristae (m). Large spherical euchromatic nucleus (N) and seemingly normal axons (A) with regular compact myelin sheath (arrows), except for few axons with broken myelin sheath (arrowheads) are also seen (×6000; scale bar: 2 µm). Prolonged darkness-ouabain group (**I**) with pyramidal cells with apparently normal organelles; rough endoplasmic reticulum (circle) and intact mitochondria (M) except for one dilated (m) as well as slight indentation of nuclear envelope (arrowheads) with few areas of condensed chromatin within the nucleus (N) (×8000; scale bar: 2 µm), (**J**) seemingly normal granular cell including regular mitochondria (M), intact rough endoplasmic reticulum (circle), and spherical nucleus (N) with well-defined nuclear envelop except for small areas of condensed chromatin (arrowhead). Axons (A) revealing regular compact myelin sheath (arrows) (×15,000; scale bar: 500 nm).

Electron microscopy examination of pyramidal cells from the control group (I) revealed a normal large triangular euchromatic nucleus and normal organelles within scant cytoplasm, mitochondria, and rough endoplasmic reticulum (RER) (Figure 8A). The granular cells revealed spherical nuclei with a dispersed distribution of chromatin and well-circumscribed mitochondria within the cytoplasm. The surrounding neuropil displayed a compact myelin sheath outlining the axons (Figure 8B). The ultrastructure of neurons in OUA-treated rats (II) was similar to that of control rats (I).

Pyramidal cells from rats subjected to chronic immobilization (IIIA) revealed extensive large areas of cytoplasmic degeneration, in addition to swollen mitochondria with destroyed cristae. The nuclei displayed highly condensed chromatin and an indented nuclear envelope (Figure 8C). The granular cells of this group revealed multiple, wide areas of cytoplasmic degeneration, marginal nuclear chromatin, and destroyed axonal myelin sheath (Figure 8D). Exposure to short photoperiods (IVA) yielded similar neuronal injury, although to a lesser extent (Figures 8E and F).

The administration of OUA to restrained rats (IIIB) caused moderate improvement in the pyramidal cells, demonstrating regular nuclei with slight condensation of chromatin, apparently normal mitochondria apart from a few swollen mitochondria and intact RER. The surrounding neuropil displayed a regular compact myelin sheath (Figure 8G). The granular cells exhibited seemingly normal architecture in the form of normal organelles within the cytoplasm with scattered dilated mitochondria, and the nucleus revealed an intact nuclear envelope. The axons exhibited a regular myelin sheath (Figure 8H).

When OUA was given to rats with exposure to short photoperiods (IVB), greater improvement was detected in the hippocampal CA3 region and dentate gyrus. The pyramidal neurons demonstrated deceptively normal cytoplasmic organelles, mitochondria, and RER apart from a few dilated mitochondria with ruptured cristae (Figure 8I). The cytoplasm of the granular cells exhibited normal organelles, mitochondria, and RER with regular spherical euchromatic nuclei as well as compact myelin sheaths, except for a few swollen mitochondria with destroyed cristae (Figure 8J).

### Histomorphometric results

Exposure to daily one-hour immobilization stress for 30 days (IIIA) revealed significant reductions (P<0.001) in the thickness of both the pyramidal layer of the CA3 zone (49.65±0.02) and the granular layer of the DG of the hippocampus (43.63±2.9) in relation to that of control rats (I) (79.34±0.35; and 71.53±1.98; respectively) and OUA-treated group (II) (78.6±0.44 and 71.53±1.98, respectively).

In addition, rats exposed to 30 days of disturbed dark/light cycles (IVA) demonstrated significant reductions (P<0.001) in the thickness of both pyramidal (50.15±0.09) and granular layers of the hippocampus (46.26±1.56), compared to control rats (I) and OUA-treated group (II).

The administration of OUA altered the stress-modulated protraction of pyramidal and granular layers, so that long-term OUA treatment to both stressed models (IIIB & IVB) re-instituted the thickness of the pyramidal layer (78.65±0.36 and 78.67±0.46, respectively) and granular layer (67.24±1.68 and 67.15±2.5, respectively), to both control (I) and the OUA-treated (II) groups, beyond those of the untreated stress models, the immobilization (IIIA) (P<0.001), and disturbed dark/light cycle (IVA) models (P<0.001).

**Figure 9:**
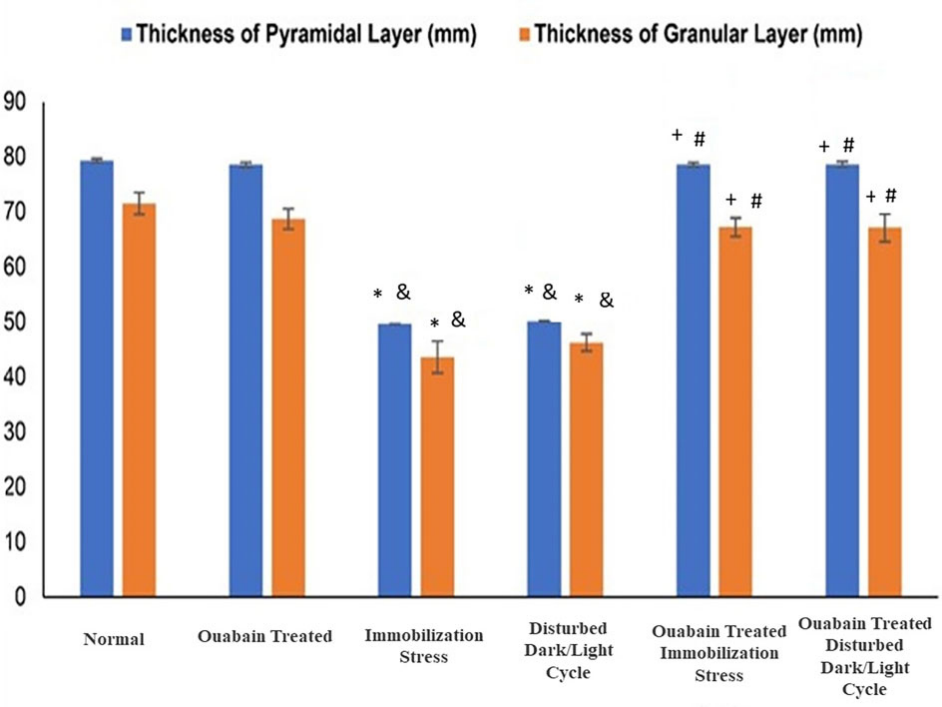
Histomorphometry of the thickness of pyramidal and granular layers in the hippocampus of study groups. Data are reported as means±SD. *P<0.05 compared to the control group; ^&^P<0.05 compared to ouabain-treated group; ^+^P<0.05 compared to untreated immobilization group; ^#^P<0.05 compared to untreated rats exposed to disturbed dark/light cycle (ANOVA).

Administration of OUA to rats (II) did not change the thickness of either the pyramidal or granular layers compared to the control group (I) (Figure 9).

### Percent body weight changes

By the end of the first 10 days, immobilized rats (IIIA) showed significantly higher gain in body weight than control rats (I) (24.22±8.48 *vs* 1.11±2.49 g, respectively; P=0.045). Conversely, no significant difference was noticed between rats subjected to disturbed dark/light cycle (IVA) and control rats (I) by the end of 10 days.

By the end of 20 days, rats submitted to the increased dark cycle (IVA) had a significantly higher weight loss relative to OUA-treated rodents (II) (-7.43±4.11 *vs* 12.32±3.02 g, P=0.017), but not controls (I). The extent of weight loss in untreated rodents in prolonged darkness (IVA) was much less than the weight gain of untreated immobilized rats (IIIA) (22.57±6.32 g, P<0.001).

At day 30, neither of the two stress models (IIIA and IVA) showed significant differences compared to control (I) and OUA-treated rats (II), although the weight loss in rats exposed to prolonged darkness (IVA) (-3.09±6.10 g) was still much lower than the weight gain in mobility-restricted rats (IIIA) (42.60±11.15 g, P=0.002).

Of interest, OUA decelerated both the restraint-related body weight gain as well as the prolonged darkness-related weight loss, which could be inferred from the lack of a significant difference with controls (I) and OUA-treated groups (II), as mentioned before.

On the other hand, 10-day OUA treatment resulted in significantly different weight loss in rats submitted to short photoperiods (IVB) compared to the weight gain in the untreated restrained rats (IIIA) (-4.27±13.35 *vs* 24.22±8.48 g, P=0.043). This discrepancy was consistent also after 20 (-1.67±2.28 *vs* 22.57±6.32 g, P=0.018) and 30 days (-1.67±2.28 *vs* 42.6±11.15 g, P=0.002), as was in the untreated models (IIIA & IVA).

**Figure 10:**
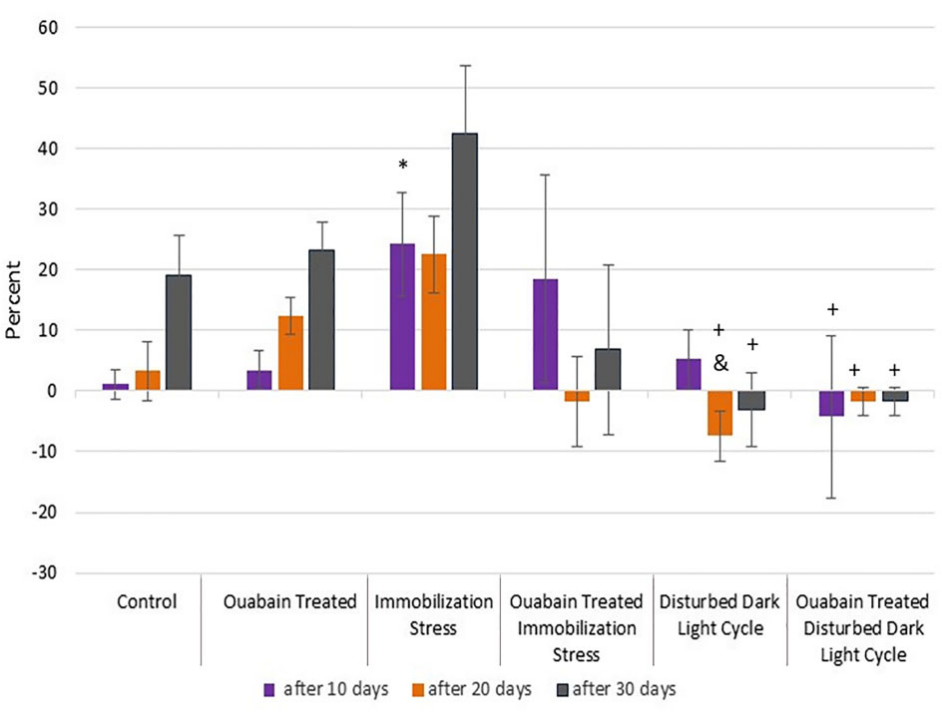
Percent changes in body weight after 10, 20, and 30 days in the study groups. Data are reported as means±SD. *P<0.05 compared to control group. ^&^P<0.05 compared to OUA-treated group. ^+^P<0.05 compared to untreated immobilized rats (Kruskal-Wallis test)

Chronic administration of OUA to rats (II) did not result in significant variation compared to control rats (I) at 10, 20, and 30 days (Figure 10).

No significant correlation was detected between percent body weight changes and the measured biochemical indices. Data not shown.

### Brain-to-body weight ratio

Regardless of the histopathological changes in the hippocampus, whether normal, neurodegeneration, or partially recovering from neurodegeneration, and regardless of slight or severe changes in body weight over time towards gain or loss, differences in the brain-to-body weight ratio among the groups were not significant and did not reflect such discrepancies. Data not shown.

### General characteristics

**Figure 11:**
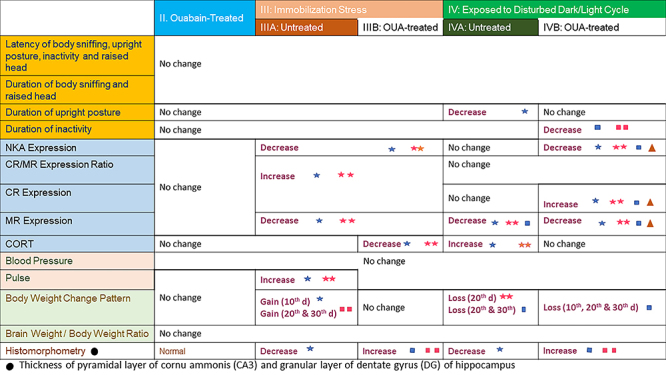
Descriptive results of behavioral, biochemical, cardiovascular, percent changes in body weight, brain-to-body weight ratio, and histomorphometry results of studied adult male Wistar albino rats (7-8 months of age), subdivided into four main groups: I: Control; II: Ouabain (OUA)-treated; III: Immobilization; and IV: Disturbed dark/light cycle. Both stress models (III and IV) were further subdivided into A: Untreated and B: Ouabain-treated. Significance: Blue star: P<0.05 compared to control group; Double pink stars: compared to OUA-treated control group; Blue square: P<0.05 compared to untreated immobilized rats; Double pink squares: P<0.05 compared to untreated disturbed dark/light cycle; Orange triangle: P<0.05 compared to ouabain-treated immobilized rats.

Descriptive summary of behavioral, biochemical, cardiovascular, percent of body weight changes, brain-to-body weight ratio, and histomorphometry results of study groups are shown in Figure 11.

## Discussion

Exposure to stress is an integral part of living creatures. Stress-evoked behavior impacts social, physiological, and many other aspects of life. Interactions to stress can be adaptive as well as potentially devastating.

A detailed literature search revealed no similar experiment comparing behavior, cardiovascular changes, hippocampal *NKA* expression, *CR/MR* expression ratio, CORT level, and histopathology of rats following immobilization or exposure to prolonged darkness. Addressing each stressor and biochemical parameter individually has provided conflicting results.

Rats facing two different types of stressors exhibited different behavioral patterns. Rats submitted to immobilization did not show any change in reaction, while those subjected to prolonged darkness showed a significant decline in the upright attack posture duration, suggestive of submission, as previously observed (24).

The fact that the restrained rats did not express behavioral changes in the presence of disordered brain chemistry and neuropathologic features could raise a ‘red flag' sign for seriousness. This occurs in humans that manifest apathy with progressive neurological disorders, such as dementia (25), emphasizing that the lack of behavioral reactions might be associated with more severe disease stage. That is to say, a ‘muffled behavior’ is as pathological as a ‘loud one’ (2).

Moreover, restrained rats had a tendency to gain weight, and this was significant in the initial 10 days of stress exposure. Despite the apparent weight loss of rats facing short photoperiods, it was non-significantly different from control rats.

Blood pressure did not show any significant variations among stressed groups, indicating that stress need not be an inevitable pre-requisite for cardiovascular disorders. Nonetheless, only restrained rats experienced increased pulse rate, although within normal physiologic rate, as an expected drawback of stress exposure (19).

The immobilization-related responses, except for the significant transient weight gain, were accompanied by extensive hippocampal *NKA* downregulation and increased *CR/MR* ratio, resulting from both upregulated *CR* and reduced *MR* expression. The concurrent failure to increase glucocorticoids might be responsible for the reduced behavioral responses and the unsustained weight gain pattern, validating the hypothalamic-pituitary-adrenal axis (HPAA) activation hypothesis of stress-related responses, with increased glucocorticoids needed to meet the metabolic demands for physiological and psychological stress reactions (26). This offers a rationale for the ‘habituation' issue that might follow repeated restraints in rats, hindering behavioral responses. Unlike the upregulation of hippocampal *CR* in our study, the behavioral tolerance occurred parallel to reduced levels of hippocampal *GR* (27), indicating the bidirectional effects of stress - stimulated HPAA leading to either the occurrence or deficiency of stress responses (28), even though the effect of immobilization on body weight remains controversial, ranging from increase, to no change, to loss (29).

Conversely, rats confronted with prolonged darkness, together with increased hippocampal CORT complying with the stress-activated HPAA hypothesis, were able to express a non-defensive response. Nevertheless, not only the lack of weight gain, but also the propensity to lose weight, though non-significant relative to controls, could indicate the existence of other mechanisms involved in stress-provoked responses, especially when the higher hippocampal CORT was driven by a significant hippocampal *MR* downregulation. In prolonged darkness, rats might be vulnerable to prolonged melatonin nocturnal surge, with subsequent MR downregulation (30), explaining the melatonin ability to promote weight loss in rodents by reducing fat deposition (31) and emphasizing the ability of melatonin to hamper the CR upregulation as one component of the stress-provoked HPAA (32). Subsequently, as was demonstrated in our work, stress can lead to reciprocal changes in one or more aspects of the day and night hormones, regardless the nature of the stressor. Besides, in terms of restraint and long nights stressors, the involvement of hippocampal *MR* might be a compulsory repercussion, but not hippocampal *NKA*, *CR*, or CORT.

The degree of biochemical diversification observed with each distressed group was consistent with the extent of neurodegenerative changes found in the hippocampal CA3 region and DG, more conspicuous in the restrained rats than in rats subjected to short photoperiods.

The role of stress in triggering hippocampal neurodegeneration is undisputed. In our rodent model, the severity of neuronal damage was not uniform between the two stressors, such that broader biochemical dysregulation in the immobilization model yielded more neuronal loss than the prolonged darkness exposure. This was, to some extent, formerly linked to hippocampal melatonin downregulation and reduced NKA activity (33), while the *CR* overexpression was regarded as a compensatory mechanism to such neurodegeneration, with loss of GC-containing neurons in decaying hippocampal cells, in an attempt to re-establish the GC signaling (34). This justifies the defective CORT increase, observed after a 21-day immobilization of rats in other experiments and in post-mortem brain specimens of patients with post-traumatic stress disorder (5).

Because an optimal dark/light cycle is required for healthy neural regeneration, prolonged darkness induced hippocampal neurodegenerative changes. In his review, González (35) highlighted the crucial role of exposure to normal circadian rhythms in mammals to prevent neurodegeneration by regulating the expression of neuroprotective proteins.

To assess the extent of involvement of *NKA* and glucocorticoids as part of a stress-elicited HPAA, as well as *MR*, and the chronic stress-related responses, we treated our rats with chronic OUA, a NKA inhibitor, at a small dose, for 30 days. The OUA effect on rats' behavior changed according to the stressor in context. During restraint exposure, daily OUA injections did not alter stress responses compared with the untreated restrained animals.

On the contrary, chronic OUA with maintained exposure to prolonged darkness reverted the accepting attitude to a hostile-like posture, manifested as a shorter duration of inactivity. From the behavioral aspect, this response seems to contrast with the calming effect seen in Wistar rats treated with lithium, another NKA inhibitor (36). This suggests that OUA might act differently than conventional NKA suppression during stress.

OUA failed to significantly alter the patterns of weight changes associated with each stressor, whether weight gain or loss, despite attempting to decelerate such stress-related phenotype.

In our experiment, the chronic small dose of *ip* OUA dissolved in distilled water did not modify rats' BP or HR, regardless of the type of stress. In contrast, chronic repeated *ip* OUA administration dissolved in saline at a much higher dose (22) and for a longer period of 6-8 weeks resulted in hypertension (37), indicating that this effect may not necessarily occur with every administration of OUA (38).

A chronic small *ip* dose of OUA did not significantly impact the biochemical features of the restraint group, apart from a pronounced reduction of hippocampal CORT in the treated restraint model, but with no obvious effect on the behavioral or the cardiovascular responses, indicating a probable defective HPAA activation and justifying the inability of OUA to overcome the non-reactive behavior in various stress responses.

The OUA reverted submissive behavior to hostile behavior during prolonged darkness, which was supported by an extensive biochemical diversification, similar to the immobilization model, except for the lack of both reduced CORT and increased *CR/MR*.

With normal CORT and upregulated *CR* during short photoperiod stress, the ability of chronic low-dose OUA to significantly suppress Na^+^/K^+^-ATPase expression, unlike the untreated model, could be due to enhanced glucocorticoid signaling, thus increasing OUA binding, as previously shown in the mammalian brain (39). Kinoshita et al. (12), however, found that a single intracerebroventricular injection of the same OUA dose caused no hippocampal *NKA* inhibition, but an increase in the Na^+^/K^+^-ATPase signaling cascade.

The histopathological assessment disclosed the neuro-regenerative potential of OUA, as evidenced by partial reversal of stress-induced structural neurodegenerative changes, such as less reduced axonal and cytoplasmic organelles, less nuclear destruction, and increased thickness of the pyramidal and granular layers. The anti-degenerative effect of OUA was consistent with results from neuronal cell culture (12) and *ip* injection of 1 μg/kg OUA into mice for 40 days after traumatic brain injury (16). This incomplete reversibility of pathological hippocampal changes, particularly in the CA3 zone, suggested resistance, especially with continued exposure to stress stimuli. The ability of OUA to enhance brain cell regeneration, even when *NKA* is downregulated, suggests that the regenerative capacity of low-dose OUA bypasses NKA targeting (40).

### Conclusion

In the current pre-clinical study, the type of stressor was critical for the extent of OUA involvement, hippocampal *NKA* expression, and *CR/MR* expression ratio. While restrained rats showed no behavioral reactivity despite hippocampal neurodegenerative changes, increased pulse rate, and overt implications of *NKA*, *CR/MR* prolonged darkness resulted in submissive posture with increased CORT, *MR* downregulation, and less neuronal damage. Our work tracked the dynamics of chronic low-dose OUA when administered to immobilized rats, with no solid behavioral or biochemical effects, apart from lowering CORT. This was in contrast to exposure to short photoperiods, in which OUA reverted the behavior to an apparent aggressive behavior involving *NKA*, *CR*, and *MR*. OUA partially reverted hippocampal neurodegeneration. Thus, it could be concluded that the effects of OUA go beyond *NKA* expression. Stress- and OUA-mediated effects over rats' BP and HR were negligible.

Further experimental and clinical work are required to discover novel mechanisms for a variety of stress situations, with the goal of optimizing a “personalized therapeutic plan” based on the stressor in context.
